# The Role of Emotion Regulation and Awareness in Psychosocial Stress: An EEG-Psychometric Correlational Study

**DOI:** 10.3390/healthcare12151491

**Published:** 2024-07-27

**Authors:** Roberta A. Allegretta, Katia Rovelli, Michela Balconi

**Affiliations:** 1 International Research Center for Cognitive Applied Neuroscience (IrcCAN), Università Cattolica del Sacro Cuore, 20123 Milan, Italy; katia.rovelli@unicatt.it (K.R.); michela.balconi@unicatt.it (M.B.); 2 Research Unit in Affective and Social Neuroscience, Department of Psychology, Università Cattolica del Sacro Cuore, 20123 Milan, Italy

**Keywords:** stress, EEG, emotion regulation, interoception, mindfulness, awareness

## Abstract

Background: In stressful situations, to overcome unpleasant emotions, individuals try to manage stress through emotion regulation strategies such as cognitive reappraisal, interoception, and mindfulness. Method: 26 healthy adults underwent a modified version of the Trier Social Stress Test (named the Social Stress Test, SST) while their electrophysiological (EEG) activity was monitored. Participants also completed self-report questionnaires prior to this, including the Five-Facet Mindfulness Questionnaire (FFMQ), Multidimensional Assessment of Interoceptive Awareness (MAIA), Emotional Regulation of Others and Self (EROS), and the Interpersonal Reactivity Index (IRI). Three brain regions of interest (ROIs) were considered in the EEG data processing: frontal, temporo-central, and parieto-occipital. Correlational analyses were performed between psychometric scales and EEG band power spectral values for each ROI. Results: The results showed positive correlations between interoceptive awareness, mindfulness, and high-frequency EEG bands (beta, alpha, gamma) over frontal ROI, indicating enhanced cognitive processing and emotional regulation. Conversely, emotion regulation and empathy measures correlated positively with low-frequency EEG bands (delta, theta), associated with improved social cognition and top-down regulatory processes. Conclusions: These findings suggest that EEG correlations of the stress response are connected to emotion regulation mechanisms, emphasizing the importance of body state awareness in managing stress and emotions for overall well-being and quality of life.

## 1. Introduction

Across the lifespan, individuals may be subjected to stressful events, which can be difficult or easy to manage depending on individual differences and coping strategies. Stress is referred to as an organism’s internal state involving physiological, cellular, and emotional reactions [1] characterized as a condition in which the organism’s homeostasis is either perceived as threatened or actually threatened by external or internal factors [2]. Unpleasant emotional sensations are typically present when under stress, including anxiety, anger, sadness, envy, jealousy, fright, guilt, and shame [3]. A wide repertoire of physiological and behavioral adaptive responses is needed to restore homeostasis. 

Among behavioral responses to restore homeostasis and manage stress, there is so-called emotion regulation, defined as the diverse range of mechanisms used to control internal emotional fluctuations by which people adjust how they feel, how they react to feelings, or how they respond to situations that elicit emotions to satisfy external demands (such as not showing negative emotions during a job interview) [4,5] and through which emotions are self-regulated, encompassing variations in emotion dynamics, latency, rise time, intensity, duration, and offset of reactions in behavioral, experiential, and physiological domains [6]. Among the most well-known emotion regulation strategies are cognitive reappraisal, emotion suppression, and mindfulness [7].

In the classical approach to the study of emotion regulation, cognitive reappraisal is defined as an antecedent-focused approach that entails interpreting a scenario that may elicit strong emotions in a way that modifies the circumstance’s emotional impact before it has fully occurred [8]. Reappraisal has been shown to successfully reduce negative affect in a variety of emotionally charged situations without a significant physiological cost, suggesting that the strategy has little to no adverse side effects [9]. On the other hand, emotion suppression is a response-focused tactic that entails the deliberate inhibition of current emotion-expressive behavior [10], but it has been demonstrated to have a significant negative impact on cognition, physiology, and interpersonal functioning in addition to failing to offer any subjective relief from the experience of negative emotions [11,12,13]. Finally, mindfulness has been defined as paying attention in a specific and purposeful way, in the present and without judgment [14], and requires intentionally focusing one’s attention on the present moment while adopting an accepting attitude toward the experience, characterized by openness and curiosity [15]. Practicing mindfulness can help people express and engage with their emotions in a healthy way, preventing issues like emotion dysregulation and under- or over-engagement [7,16].

However, more recently a novel approach has taken into consideration a crucial aspect that might influence emotion regulation processes: *interoception* [17,18]. Indeed, at the very core of the concept of mindfulness, there is interoception*,* considered the primary mechanism by which one benefits from the mindfulness practice. Interoception can be defined as the ability to perceive internal bodily sensations [18]. Notably, it has been demonstrated that mindfulness modulates and has a neuroplasticity effect not only on the default mode network (DMN) [19] but also on the interoceptive network, including the sensorimotor cortex, inferior frontal gyrus, cingulate cortex, and insular cortex [20,21,22,23]. Additionally, research suggests that better emotion regulation is correlated with better interoceptive skills [20,24,25]. These findings have prompted a general conceptualization of emotion suppression as a “maladaptive” regulatory strategy and cognitive reappraisal and mindfulness as “adaptive” techniques [8].

Interestingly, emotion regulation and its “adaptive” strategies require a certain level of awareness, declining in different but related aspects: emotional and interoceptive. Notably, emotional awareness (EA) is defined by Lane and Schwartz [26] as the capacity to recognize, categorize, and express one’s feelings as well as those of others. Reappraisal and high EA can help mitigate the potentially harmful effects of unpleasant emotions on the processing of emotional information [27]. Instead, interoceptive awareness (IA) refers to the conscious experience of internal bodily sensations that contribute to the perception of one’s physiological state. These sensations include heart rate, breathing, and autonomic nervous system feelings associated with emotions [28,29]. Both EA and IA require high levels of focused attention, attentional control, and regulation [30].

Usually, to assess all these emotion regulation-related aspects (i.e., cognitive reappraisal, mindfulness, and interoception), both clinicians and scholars use self-report scales, each assessing and focusing on specific aspects of the phenomenon. Specifically, the tools that are frequently used to measure interoception and mindfulness are the Five-Facet Mindfulness Questionnaire (FFMQ) [31] and the Multidimensional Assessment of Interoceptive Awareness (MAIA) [30]. On the other hand, the Emotion Regulation of Others and Self scale (EROS) [32] is often used to investigate and assess emotion regulation. Notably, the ability to regulate not only one’s emotions but also other people’s emotions and feelings requires a certain level of emotional intelligence, which, in turn, also needs empathy and the understanding of other’s thoughts. The latter aspects are usually assessed via the Interpersonal Reactivity Index (IRI) [33].

Recent studies have examined psychosocial stress, which arises from social interactions due to their novelty, unpredictability, uncontrollability, or the involvement of social-evaluative risks [34]. This stress results from cognitive appraisal exceeding an individual’s coping capacity. Notably, researchers have identified three phases in the acute stress response to psychosocial stressors: the *anticipatory* phase (awareness of an impending stressor), the *reactive* phase (direct interaction with the stressor), and the *recovery* phase (post-stressor exposure) [34,35]. Each phase involves distinct physiological and psychological changes driven by the underlying stress response.

Studies about psychosocial stress primarily focus on the brain, due to its crucial function as the organ responsible for coordinating the many stages of the psychosocial stress response [36,37] through the use of neuroscientific tools such as electroencephalography (EEG). EEG is a non-invasive method that records minute variations in electrical potential caused by cortical neurons’ activity in real time with a temporal precision of milliseconds using electrodes applied to the scalp. Additionally, brain activity associated with particular perceptual, cognitive, and affective processes can be studied and investigated through the analysis of frequency bands (delta, theta, alpha, beta, and gamma), as well as through the investigation of their localization on the scalp and their functional meaning. 

Going down to specifics, low-frequency oscillations of delta and theta are associated with motivational and emotional processes [38] that are important for preparing the body to react to stressful events, especially when they call for introspection and independent emotion control. Notably, delta waves are linked to emotional regulation and response planning, and they are particularly active in the frontal and temporo-central areas under stressful situations [39] to effectively support emotional management under pressure and are essential for coping mechanisms during challenging circumstances. Theta waves, on the other hand, are essential in top-down regulation processes including awareness, sustained and mindful attention, inhibition and cognitive control [38], and problem-solving abilities [40], especially in the parieto-occipital region.

Regarding the alpha band, studies showed a drop in alpha power in response to psychosocial stress and under various stress stages [41]. This decrease in alpha power is thought to be connected to elevated cortical activity, which is indicative of the brain’s inhibitory control system, which plays a supportive role in coping with stress. A condition of increased alertness and vigilance brought on by psychosocial stress activates the hypothalamic–pituitary–adrenal axis (HPA), increases cortical activity, and lowers frontal alpha power [42].

Stress-related cognitive functions including self-regulation and attention have been linked to beta activity, especially in the frontal area [43]. Increased beta power during stress responses has been linked to continued cognitive processing and the activation of brain networks to address psychosocial stressors [44]. Finally, numerous cognitive processes, including focused attention, elevated arousal, top-down attentional mechanisms, and conscious awareness, have been linked to gamma oscillations [45,46]. Research has indicated that after being exposed to emotional stimuli, gamma band activity is associated with cognitive reappraisal, especially in the left frontal and parietal regions [47]. 

However, emotion regulation, which has traditionally been investigated using resting alpha frontal EEG asymmetry (a trait measure), presents a challenge because emotion regulation is inherently dynamic. To address this, Coan et al. [48] proposed the Capability Model, suggesting that while the dispositional model of frontal EEG asymmetry assesses an individual’s approach versus withdrawal disposition regardless of the situation, the capability model measures the extent to which individuals can engage in approach or withdrawal responses, or inhibit those responses, depending on situational demands. According to this perspective, state measures of the neural correlates of emotion regulation might better explain the variability in behavioral outcomes during emotional challenges compared to trait measures [48]. Therefore, the employment of EEG involving not only frontal alpha activity but also other EEG bands during a stressful task in conjunction with a psychometric assessment of emotion regulation and its associated strategies might offer a comprehensive methodology to better understand and examine the phenomenon of emotion regulation in the context of psychosocial stress dynamics. To the best of our knowledge, at this time, there are no studies that have investigated correlations between situational measures of emotion regulation under stress and psychometric measures of emotion regulation strategies, including cognitive reappraisal, mindfulness, and interoception. Based on this theoretical background and due to the existing gap in the literature, the current work aims to provide further insight into the understanding of psychosocial stress dynamics, individual differences, and patterns of neural activation. Therefore, to understand if and how (i.e., in a direct or inversely way) EEG correlates of psychosocial stress responses are correlated to emotion regulation, including cognitive reappraisal, interoception, and mindfulness, this study will adopt a novel approach and methodology by employing a combination of experimental tasks designed to induce psychosocial stress (SST) and self-report measures to assess emotion regulation strategies.

To pursue this aim, participants’ EEG activity was continuously recorded during a modified version of the Trier Social Stress Test (TSST) [49], named SST (Social Stress Test). Indeed, when studying stress and its related induced responses, scholars have mostly used the Trier Social Stress Test (TSST) created to elicit a psychobiological stress response [50], consisting of a spontaneous speech that must be completed in front of an audience. Before the beginning of the SST, participants fulfilled the FFMQ, MAIA, EROS, and IRI. Based on the aforementioned theoretical and methodological background, we hypothesized we would find different patterns of correlation between the psychometric measures and EEG correlates of activation during the SST. Specifically, we expected to find positive correlations between the psychometric measures of interoceptive awareness (MAIA) and mindfulness (FFMQ) and the high-frequency bands (beta, alpha, and gamma). Indeed, the concepts of interoception and perception are central to understanding one’s own body and external stimuli, requiring a high level of awareness of bodily sensations and attention. Therefore, we expect (i) an associated higher level of beta in frontal areas since increased beta power in these areas during stress responses has been linked to continued cognitive processing [44] and improved focus and attention to body sensations, promoting emotional awareness and self-regulation [51]; (ii) an associated increased activity of alpha in the temporal area since studies have shown how a higher level of mindfulness is linked to an enhanced capacity to use sensory alpha modulation to facilitate behaviourally relevant internal stimuli by raising alpha activity to block competing sensory processes [52]; and (iii) an associated enhancement of gamma band activity in frontal areas since mindfulness has been found to increase gamma band activity in these areas, reflecting improved emotional regulation [53] and cognitive reappraisal after exposure to positive, negative, or neutral stimuli [45,54].

On the other hand, we expected to find positive correlations between the psychometric measures of emotion regulation (EROS) and empathy (IRI) and the low-frequency bands (delta and theta) in temporal areas. Indeed, delta activity rises during social judgment paradigms, especially when one is responding to social context feedback [55]. Furthermore, pro-social emotions toward others and enhanced empathy for fictional characters have been linked to higher delta activity [38,56], whereas a range of behavioral and affective characteristics, as well as high-level processes like sustained attention, inhibition, and cognitive control, have been linked to the theta frequency [38]. Finally, greater theta activity is associated with enhanced awareness, clarity, acceptance, inhibitory control, and flexibility in emotion regulation strategies [57]. 

## 2. Materials and Methods

### 2.1. Sample

The sample included 26 healthy adults (Mean age = 23.04, SD age = 1.45, age range: 22–28, N_male_ = 10, N_female_ = 16). 

The criteria for inclusion were individuals aged between 18 and 32 years with normal or corrected visual and auditory acuity. The criteria for exclusion were established to confirm the participation of healthy adults, excluding any factors that could potentially influence the study’s results. Firstly, individuals with a past medical history of neurological or psychiatric disorders were excluded. This encompassed any diagnosed conditions affecting the brain or nervous system, along with any mental health disorders. Secondly, participants who were concurrently undergoing therapies involving psychoactive drugs that could impact central nervous system functioning were also omitted. This included medications such as antidepressants, antipsychotics, and anxiolytics, among others. Lastly, individuals exhibiting clinically significant distress or a history of burnout were barred from participation. This included individuals experiencing substantial stress or emotional distress, as well as those with a history of burnout or chronic stress. 

To validate low-stress levels in the preceding thirty days, each participant completed the Italian version of the Perceived Stress Scale-10 (PSS-10) [58]. For each participant, the total score was either equal to or less than 27.

Written informed consent was obtained from all participants without any form of compensation. The study was approved by the Ethics Committee of the Department of Psychology, Catholic University of The Sacred Heart, Milan, Italy, and was conducted in adherence to the guidelines outlined in the Declaration of Helsinki (2013) and according to the GDPR–Reg. UE 2016/679 and its ethical guidelines. 

### 2.2. Experimental Procedure and Psychometric Self-Report Scales: FFMQ, MAIA, EROS, and IRI

The experiment was conducted in a laboratory setting. Participants were preliminarily informed about the nature of the experiment. It was explicitly communicated to them that this was not a real job interview aimed at employment but rather a simulation of a job interview conducted in a digital format. The participants were also made aware that they would be evaluated by a fictitious examination panel, and that the sessions would be video recorded. They were also informed that the procedure would involve the application of a monitoring device, specifically a non-invasive 18-channel EEG. After signing the informed consent for participation in the research and having the data handling procedures explained, each participant was assessed in a controlled environment room (e.g., moderately lit and with noise control) equipped with a station consisting of a desk, a monitor, and a chair. Participants were instructed to position themselves approximately 100 cm away from the monitor and ensure that their feet were firmly planted on the ground for the duration of the experiment.

Before the beginning of the Social Stress Test, all participants were asked to complete a series of questionnaires to further evaluate their psychological and emotional states: the FFMQ, the MAIA, the EROS, and the IRI.

*Five-Facet Mindfulness Questionnaire (FFMQ)*. The FFMQ [31,59] is a psychometric instrument used to assess an individual’s level of mindfulness, which refers to their ability to be fully present and engaged in the current moment, without judgment or distraction. The questionnaire was developed through a factor analysis of existing mindfulness measures and consists of 39 items, each rated on a five-point Likert scale. This scale ranges from “1” (indicating never or very rarely true) to “5” (signifying very often or always true). The FFMQ measures five different facets of mindfulness, including observing, describing, acting with awareness, non-judging, and non-reactivity. The FFMQ evaluates five key aspects of mindfulness: (i) Observing (i.e., the ability to notice or attend to internal and external stimuli, such as bodily sensations, emotions, thoughts, and environmental sounds), (ii) Describing (i.e., the capacity to label and put into words internal experiences), (iii) Acting with awareness (i.e., the degree of attention and focus given to one’s actions in the present moment, avoiding automatic or habitual behavior), (iv) Non-judging (i.e., the ability to accept one’s thoughts and feelings without judgment or self-criticism), and (v) Non-reactivity (i.e., the ability to let thoughts and emotions come and go without becoming overwhelmed or reacting impulsively). 

*Multidimensional Assessment of Interoceptive Awareness (MAIA)*. The MAIA [30,60,61] is a self-report measure designed to assess individuals’ awareness of their internal bodily sensations. The MAIA consists of 32 items, each rated on a six-point Likert scale, ranging from 0 (Never) to 5 (Always). The items are distributed across eight distinct scales: (i) Noticing (i.e., the tendency to pay attention to uncomfortable, comfortable, and neutral body sensations); (ii) Not-Distracting (i.e., the ability to maintain attention on bodily sensations without becoming distracted); (iii) Not-Worrying (i.e., the propensity to not experience emotional distress or worry as a result of focusing on bodily sensations); (iv) Attention Regulation (i.e., the capacity to actively regulate and control attention towards body sensations); (v) Emotional Awareness (i.e., recognition of the connection between body sensations and emotional states); (vi) Self-Regulation (i.e., the utilization of body sensations to facilitate emotional self-regulation); (vii) Body Listening (i.e., actively listening to the body for insight and understanding); and (viii) Trusting (i.e., the belief in the accuracy and reliability of body sensations as indicators of one’s internal state). 

*Emotional Regulation of Others and Self (EROS)*. The EROS scale [32] is designed to assess an individual’s ability to regulate their own emotions, as well as their ability to influence the emotions of others. The scale consists of 19 items, each rated on a five-point Likert scale. This scale ranges from “1” (indicating never) to “5” (indicating always). The scale is composed of four distinct subscales, each targeting a specific aspect of emotional regulation: (i) Extrinsic affect worsening (i.e., the tendency of an individual to worsen the emotional state of others extrinsically, which assesses behaviors such as provoking negative emotions in others, exacerbating existing negative emotional states, or undermining others’ emotional well-being through external means); (ii) Extrinsic affect improving (i.e., an individual’s ability to improve the emotional state of others extrinsically, which examines behaviors such as providing comfort, offering positive feedback, or using external strategies to enhance others’ emotional well-being; (iii) Intrinsic affect worsening (i.e., the tendency of an individual to worsen their own emotional state intrinsically, which evaluates behaviors such as rumination, self-blame, or other intrinsic strategies that may exacerbate negative emotional states); (iv) Intrinsic affect improving (i.e., an individual’s ability to improve their own emotional state intrinsically, which measures strategies such as cognitive reappraisal, self-soothing, or other intrinsic methods used to enhance one’s own emotional well-being).

*Interpersonal Reactivity Index (IRI)*. The IRI [62] is a psychological measure designed to assess the multidimensional construct of empathy. This self-report instrument aims to evaluate an individual’s dispositional tendency to perceive and experience others’ emotions, thoughts, and feelings, as well as their subsequent propensity to respond with appropriate affective and behavioral reactions. Participants are instructed to indicate the degree to which they agree or disagree with each statement using a 5-point Likert scale, ranging from “1” (indicating ‘Does not describe me well’) to “5” (indicating ‘Describes me very well.’). The IRI comprises 28 items, distributed evenly across four distinct subscales: (i) the Perspective Taking subscale (i.e., the individual’s propensity to adopt others’ psychological viewpoints spontaneously); (ii) Fantasy (i.e., the respondent’s inclination to transpose themselves imaginatively into the experiences of fictional characters in literature, movies, or plays); (iii) Empathic Concern (i.e., the extent to which an individual experiences feelings of warmth, compassion, and concern for others in distress); (iv) and Personal Distress (i.e., the individual’s tendency to experience discomfort, anxiety, and unease in response to others’ negative emotions).

### 2.3. The SST Task

Upon fulfillment of the psychometric self-report scales and correct positioning, EEG electrodes were applied to each participant’s scalp, and the baseline was recorded with eyes open and closed for a total duration of 120 s per condition.

**Figure 1:**
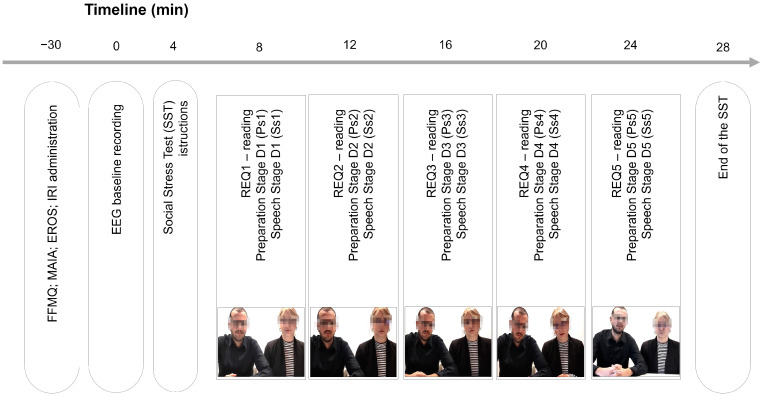
Sequence of the discourses (D1-5) within the experimental procedure. For every discourse, the following sequence was reported: (i) the reading of the request (REQ); (ii) the Preparation Stage (Ps), with a maximum time of 120 s; and (iii) the Speech stage, with a maximum time of 60 s (Ss). EEG activity was monitored from the baseline throughout the SST. The psychometric tests (FFMQ, MAIA, EROS, and IRI) were administered at the beginning of the experimental procedure.

Subsequently, the Social Stress Test (SST) started. The experimental procedure lasted approximately 30 min (Figure 1). 

The SST was administered via a web-based experiment management platform (Qualtrics XM platform; Qualtrics LLC, Provo, UT, USA). 

In the SST, participants were required to prepare (i.e., Preparation Stage, “Ps_1÷5_”; maximum preparation time 120 s) and then deliver (i.e., Speech Stage, “Ss_1÷5_”; maximum exposure time 60 s) five separate speeches on topics with progressively increasing emotional demands (REQ1÷5. REQ1: “*prepare the best presentation of yourself*”; REQ2: “*describe a situation where you encountered difficulty in making a decision*”; REQ3: “*describe a situation where you made a difficult decision without any support*”; REQ4: “*describe a situation where you made a decision taking full responsibility on behalf of others, without being able to consult them*”; REQ5: “*describe a situation where you made a decision in disagreement with the rest of the group*”). To ensure the robustness of the REQ in eliciting emotional salience, observational behavioral measures were collected and coded by external evaluators. The coding results showed that the observed behaviors were consistent with the expected emotional responses for each REQ. Furthermore, participants were exposed to five video–auditory emotional stimuli (five dynamic movies depicting an examining board commenting on their performance) with progressively higher negative emotional connotations during each discourse between the preparation and speech stages (e.g., V1: friendliness; V2: neutrality; V3: boredom; V4: increasing impatience; D5: total aversion). Each video used in the study was between 14 and 17 s long, featuring a panel of two individuals (one male and one female). Videos were presented in color. To ensure technical consistency, the five videos were processed using DaVinci Resolve (Blackmagic Design Pty Ltd.; Port Melbourne; Victoria, Australia) and Adobe Premiere Pro (Adobe; San Jose, California) to standardize the resolution (1920 × 1080), frame rate, bitrate, and format (.mp4, 16:9 aspect ratio). Audio levels were normalized and audio compression was standardized (AAC) using Audacity (Muse Group & contributors; Limassol, Cyprus). 

The stimuli used in this investigation were thoroughly examined and assessed by independent judges to ensure that they were reliable and coherent in yielding the intended experimental outcomes. Post-experiment questions were also given to participants to establish whether they were stressed about the job interview circumstances. The findings showed that 92% of participants experienced stress during the test (identifying the presence of certain physiological markers, such as sweating on the hands, a feeling of heat, an accelerated heartbeat, bewilderment or mental blankness, and trembling in the voice).

### 2.4. EEG Data Acquisition and Processing

Electroencephalographic data were collected using NEUROSCAN 4.2 software and an 18-channel DC amplifier (SYNAMPS) for the baseline, Ps1_÷5_, and Ss_1÷5_ of the SST. Using Ag/AgCl electrodes, ElectroCap was applied to 18 scalp sites in accordance with the 10/20 scheme, with reference to the earlobes (Jasper, 1958). Additionally, to prevent visual interference, two electrooculographic electrodes were positioned above and below each participant’s left eye. A 50 Hz notch input filter was used to filter the data after they were recorded at 1000 Hz. Before data collection, the electrode impedance was verified to make sure it stayed below 5 kΩ. Then, using Vision Analyzer2 software (Brain Products GmbH, Gilching, Germany), data from the baseline, Ps1_÷5_, and Ss_1÷5_ were analyzed offline (IIR bandpass filter 0.1–50 Hz, 48 dB/octave) and adjusted using an ICA-based method (Jung et al., 2000). After thorough visual inspection and EOG correction, only segments free of eye or muscle artifacts and other disruptions were taken into consideration (rejected epochs, 3%). For the subsequent statistical analysis, every electrode (AFz, Fp1, F7, F3, Fz, F4, F8, Fp2, T7, C3, Cz, C4, T8, P3, Pz, P4, O1, and O2) was utilized. To preserve the integrity of the electroencephalogram data, the data were successively epoched within a 2000 ms window. After that, condition-specific Power Spectral Density (PSD) was calculated using artifact-free data and the fast Fourier transform (Hamming window, resolution: 0.5 Hz). Finally, the average PSD was extracted for each phase taken into consideration for the major electroencephalographic frequency bands (delta: 0.5–3.5 Hz, theta: 4–7.5 Hz, alpha: 8–12.5 Hz, beta: 13–30 Hz, and gamma: 30.5–50 Hz). All task-related data were also standardized to each participant’s eyes-open baseline.

**Figure 2:**
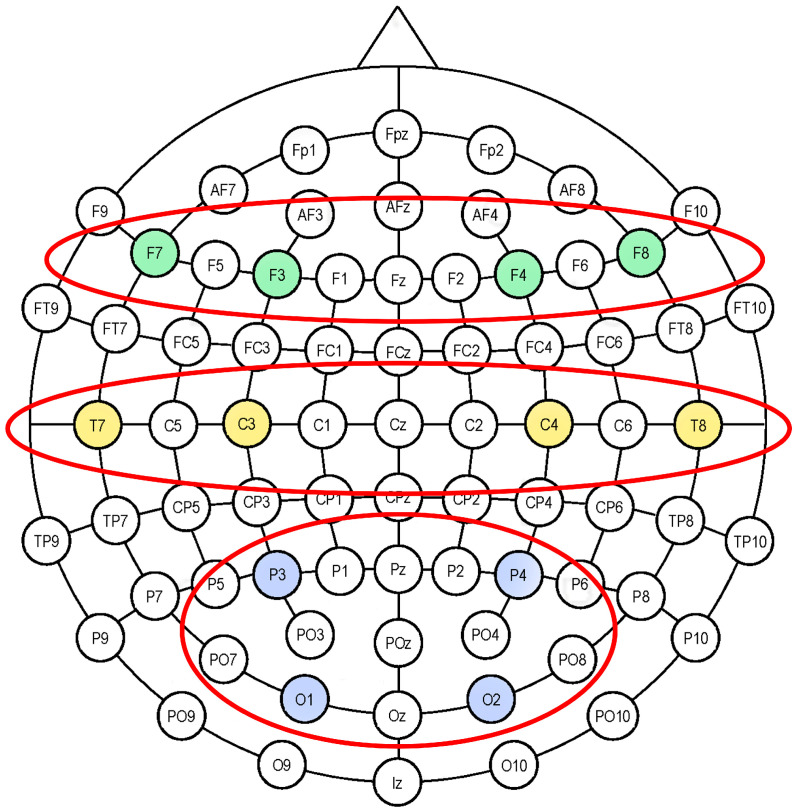
Electroencephalography (EEG). Eighteen-channel EEG montage adopted in the study, according to the 10/20 system of electrode placement (Jasper, 1958), and the selected regions of interest: frontal (ROIF: F3; F4; F7; F8), temporo-central (ROITC: T7; T8; C3; C4), and parieto-occipital (ROIPO: P3; P4; O1; O2).

Three Regions of Interest (ROIs) generated through averaging frontal (ROIF: F3; F4; F7; F8), temporo-central (ROITC: T7; T8; C3; C4), and parieto-occipital (ROIPO: P3; P4; O1; O2) electrodes were taken into consideration in the statistical analysis of the data (Figure 2).

### 2.5. Correlational Analysis

Correlation analyses (Pearson correlation coefficients) were computed between each subscale of the self-report scales utilized (FFMQ, MAIA, EROS, and IRI) and test-related changes in power spectral values for EEG bands (ROI: ROIF, ROITC; ROIPO; EEG bands: delta, theta, alpha, beta, and gamma) in each stage of the test (Ps and Ss) for the five discourses (Ps1_÷5_ and Ss_1÷5_).

## 3. Correlational Results

### 3.1. FFMQ

*Observing*. A significant positive correlation was found between Observing and ROIF in Ps_4_ for the beta frequency band (r = 0.407, *p* = 0.039) and the gamma frequency band (r = 0.400, *p* = 0.043). 

*Acting with Awareness*. For the theta frequency band, a significant positive correlation was found between Acting with Awareness and ROIPO in Ps_3_ (r = 0.538, *p* = 0.005). 

**Figure 3:**
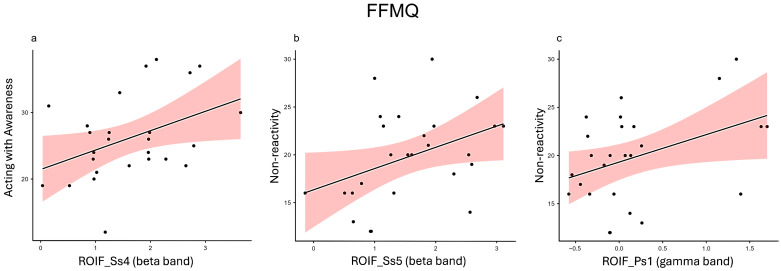
Representative scatterplots for key Pearson correlations between the FFMQ and the test-related changes in power spectral values for EEG bands (ROI: ROIF, ROITC; ROIPO). (**a**) The scatter plots display a positive correlation between the Acting with Awareness subscale and the beta power of the ROIF in Ss_4_. (**b**) The scatter plots display a positive correlation between the Non-reactivity subscale and the beta power of the ROIF in Ss_5_. (**c**) The scatter plots display a positive correlation between the Non-reactivity subscale and the gamma power of the ROIF in Ps_1_.

Furthermore, for the beta frequency band, Acting with Awareness was positively correlated with ROIF in both Ss_4_ (r = 0.409, *p* = 0.038) (Figure 3a) and Ss_5_ (r = 0.434, *p* = 0.027). 

*Non-reactivity*. Within the beta frequency band, Non-reactivity displayed several significant positive correlations with ROITC in Ps_1_ (r = 0.410, *p* = 0.038) and Ps_4_ (r = 0.517, *p* = 0.007) and in ROIF in Ss_3_ (r = 0.447, *p* = 0.022) and Ss_5_ (r = 0.392, *p* = 0.048) (Figure 3b). 

Additionally, in the gamma frequency band, Non-reactivity showed a significant positive correlation with ROIF in Ps_1_ (r = 0.404, *p* = 0.041) (Figure 3c).

### 3.2. MAIA

*Noticing*. In the gamma frequency band, Noticing showed a significant positive correlation with ROIF in Ss_1_ (r = 0.454, *p* = 0.020), Ss_3_ (r = 0.407, *p* = 0.039), and Ss_4_ (r = 0.535, *p* = 0.005). 

*Not Worrying*. In the beta frequency band, a significant positive correlation was found between Not Worrying and ROITC in Ps_1_ (r = 0.425, *p* = 0.030). 

In the gamma frequency band, Not Worrying showed positive correlations with ROIF in Ps_1_ (r = 0.521, *p* = 0.006) and in Ss_2_ (r = 0.589, *p* = 0.002) and with ROITC in Ps_1_ (r = 0.420, *p* = 0.033). 

*Attention Regulation*. Within the beta frequency band, Attention Regulation demonstrated positive correlations with ROIF in Ps_5_ (r = 0.454, *p* = 0.020), Ss_2_ (r = 0.425, *p* = 0.031), and Ss_4_ (r = 0.399, *p* = 0.044). 

In the gamma frequency band, a positive correlation was also observed between Attention Regulation and ROIF in Ps_5_ (r = 0.466, *p* = 0.016).

*Emotional Awareness*. For the beta frequency band, Emotional Awareness showed positive correlations with ROIF in Ps_5_ (r = 0.458, *p* = 0.019) and Ss_2_ (r = 0.410, *p* = 0.038). 

**Figure 4:**
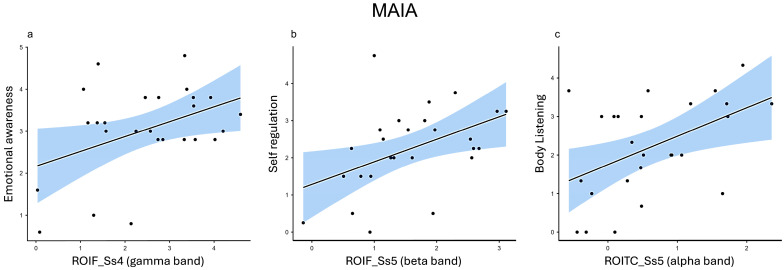
Representative scatterplots for key Pearson correlations between the MAIA and the test-related changes in power spectral values for EEG bands (ROI: ROIF, ROITC; ROIPO). (**a**) The scatter plots display a positive correlation between the Emotional awareness subscale and the gamma power of the ROIF in Ss_4_. (**b**) The scatter plots display a positive correlation between the Self-regulation subscale and the beta power of the ROIF in Ss_5_. (**c**) The scatter plots display a positive correlation between the Body Listening subscale and the alpha power of the ROITC in Ss_5_.

In the gamma frequency band, Emotional Awareness was positively correlated with ROIF in Ss_1_ (r = 0.460, *p* = 0.018), Ss_3_ (r = 0.425, *p* = 0.030), and Ss_4_ (r = 0.419, *p* = 0.033) (Figure 4a). 

*Self-Regulation*. In the beta frequency band, Self-Regulation demonstrated significant positive correlations with ROIF in Ps_5_ (r = 0.428, *p* = 0.029), Ss_2_ (r = 0.452, *p* = 0.020), Ss_3_ (r = 0.435, *p* = 0.026), and Ss_5_ (r = 0.455, *p* = 0.019) (Figure 4b) and with ROITC in Ss_5_ (r = 0.389, *p* = 0.050). 

In the gamma frequency band, positive correlations were observed between Self-Regulation and ROIF in Ss_3_ (r = 0.428, *p* = 0.029), Ss_4_ (r = 0.474, *p* = 0.014), and Ss_5_ (r = 0.411, *p* = 0.037).

*Body Listening*. In the alpha frequency band, Body Listening showed significant positive correlations with ROIF in Ss_2_ (r = 0.459, *p* = 0.018) and Ss_4_ (r = 0.388, *p* = 0.050); with ROITC in Ps_1_ (r = 0.392, *p* = 0.048), Ps_5_ (r = 0.410, *p* = 0.038), Ss_2_ (r = 0.467, *p* = 0.016), Ss_3_ (r = 0.471, *p* = 0.015), Ss_4_ (r = 0.419, *p* = 0.033), and Ss_5_ (r = 0.476, *p* = 0.014) (Figure 4c); and with ROIPO in Ps_1_ (r = 0.507, *p* = 0.008), Ps_2_ (r = 0.439, *p* = 0.025), Ps_3_ (r = 0.522, *p* = 0.006), Ps_5_ (r = 0.526, *p* = 0.006), Ss_1_ (r = 0.454, *p* = 0.020), Ss_2_ (r = 0.454, *p* = 0.020), Ss_3_ (r = 0.472, *p* = 0.015) and Ss_5_ (r = 0.389, *p* = 0.049). 

Furthermore, in the beta frequency band, Body Listening correlated positively with ROIPO in Ps_3_ (r = 0.454, *p* = 0.020). 

Lastly, in the gamma frequency band, a significant positive correlation was found between Body Listening and ROIF in Ss_4_ (r = 0.447, *p* = 0.022).

*Trusting*. In the delta frequency band, Trusting showed significant positive correlations with ROIF in Ps_3_ (r = 0.461, *p* = 0.018) and ROITC in Ps_3_ (r = 0.461, *p* = 0.018). 

In the alpha frequency band, positive correlations were observed between Trusting and ROIF in Ss_1_ (r = 0.431, *p* = 0.028) and ROITC in Ps_3_ (r = 0.396, *p* = 0.045). 

In the gamma frequency band, Trusting correlated positively with ROITC in Ss_1_ (r = 0.461, *p* = 0.018) and ROIPO in Ps_3_ (r = 0.394, *p* = 0.047).

### 3.3. EROS

**Figure 5:**
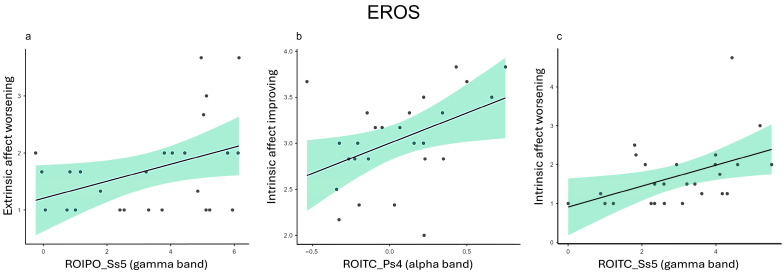
Representative scatterplots for key Pearson correlations between the EROS and the test-related changes in power spectral values for EEG bands (ROI: ROIF, ROITC; ROIPO). (**a**) The scatter plots display a positive correlation between the Extrinsic affect worsening subscale and the gamma power of the ROIPO in Ss_5_. (**b**) The scatter plots display a positive correlation between the Intrinsic affect improving subscale and the alpha power of the ROITC in Ps_4_. (**c**) The scatter plots display a positive correlation between the Intrinsic affect worsening subscale and the gamma power of the ROITC in Ss_5_.

*Extrinsic affect worsening*. In the gamma frequency band, Extrinsic affect worsening showed a positive correlation with ROIPO in Ps_5_ (r = 0.392, *p* = 0.048), Ss_1_ (r = 0.473, *p* = 0.015), Ss_2_ (r = 0.421, *p* = 0.032), Ss_3_ (r = 0.526, *p* = 0.006), and Ss_5_ (r = 0.390, *p* = 0.049) (Figure 5a), and with ROITC in Ss_3_ (r = 0.450, *p* = 0.021) and Ss_5_ (r = 0.398, *p* = 0.044). 

*Intrinsic affect improving*. Intrinsic affect improving showed a significant positive correlation with ROITC in Ps_5_ for the delta band (r = 0.448, *p* = 0.022) and the theta band (r = 0.448, *p* = 0.022). 

For the alpha band, significant positive correlations were identified between Intrinsic affect improving with ROIF in Ss_2_ (r = 0.407, *p* = 0.039) and with ROITC in Ps_4_ (r = 0.436, *p* = 0.026) (Figure 5b), Ps_5_ (r = 0.454, *p* = 0.020), Ss_2_ (r = 0.444, *p* = 0.023), Ss_3_ (r = 0.445, *p* = 0.023), and Ss_5_ (r = 0.412, *p* = 0.037). 

Additionally, Intrinsic affect improving showed a significant positive correlation with ROIF in Ps_5_ for the beta band (r = 0.462, *p* = 0.017) and the gamma band (r = 0.417, *p* = 0.034).

*Intrinsic affect worsening*. In the beta frequency band, Intrinsic affect worsening showed positive correlations with ROITC in Ss_2_ (r = 0.392, *p* = 0.047) and with ROIPO in Ss_1_ (r = 0.393, *p* = 0.047). 

In the gamma frequency band, significant positive correlations were found with ROITC in Ss_3_ (r = 0.517, *p* = 0.007) and Ss_5_ (r = 0.453, *p* = 0.020) (Figure 5c) and with ROIPO in Ps_4_ (r = 0.439, *p* = 0.025).

### 3.4. IRI

**Figure 6:**
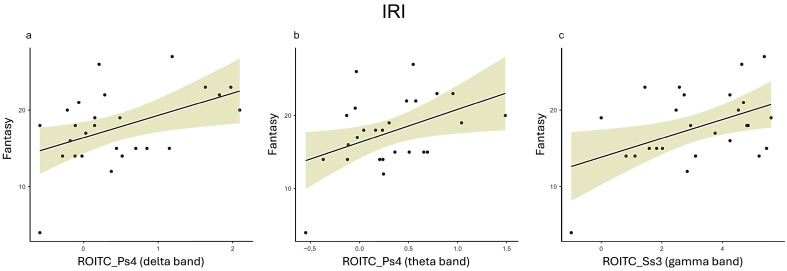
Representative scatterplots for key Pearson correlations between the IRI and the test-related changes in power spectral values for EEG bands (ROI: ROIF, ROITC; ROIPO). (**a**) The scatter plots display a positive correlation between the Fantasy subscale and the delta power of the ROITC in Ps_4_. (**b**) The scatter plots display a positive correlation between the Fantasy subscale and the theta power of the ROITC in Ps_4_. (**c**) The scatter plots display a positive correlation between the Fantasy subscale and the gamma power of the ROITC in Ss_3_.

*Fantasy*. In the delta frequency band, Fantasy showed significant positive correlations with ROITC in Ps_4_ (r = 0.463, p = 0.017) (Figure 6a) and Ss_4_ (r = 0.393, p = 0.047) and with ROIPO in Ps_4_ (r = 0.453, *p* = 0.020).

For the theta frequency band, positive correlations were observed between Fantasy and ROITC in Ps_4_ (r = 0.429, *p* = 0.029) (Figure 6b) and Ss_3_ (r = 0.467, *p* = 0.016) and with ROIPO in Ps_4_ (r = 0.475, *p* = 0.014).

In the gamma frequency band, Fantasy correlated positively with ROITC in Ss_3_ (r = 0.460, *p* = 0.018) (Figure 6c).

*Empathic Concern*. In the delta frequency band, Empathic Concern showed a significant positive correlation with ROITC in Ps_4_ (r = 0.422, *p* = 0.032).

*Perspective-Taking*. For the theta frequency band, Perspective-Taking demonstrated a positive correlation with ROITC in Ss_1_ (r = 0.399, *p* = 0.043). 

In the beta frequency band, positive correlations were found between Perspective-Taking and ROIF in Ps_4_ (r = 0.412, *p* = 0.037), Ss_1_ (r = 0.477, *p* = 0.014), Ss_2_ (r = 0.472, *p* = 0.015), and Ss_3_ (r = 0.502, *p* = 0.009). 

In the gamma frequency band, Perspective-Taking was positively correlated with ROIF in Ps_4_ (r = 0.486, *p* = 0.012) and Ss_2_ (r = 0.454, *p* = 0.020).

*Personal Distress*. In the delta frequency band, Personal Distress showed a positive correlation with ROITC in Ss_4_ (r = 0.450, *p* = 0.021). 

In the alpha frequency band, significant positive correlations were observed between Personal Distress and ROIPO in Ps_3_ (r = 0.529, *p* = 0.005) and Ss_1_ (r = 0.439, *p* = 0.025). 

In the gamma frequency band, Personal Distress correlated positively with ROIPO in Ss_1_ (r = 0.451, *p* = 0.021).

## 4. Discussion and Conclusions

The current study seeks to provide a comprehensive methodology to fill a gap and better understand and examine the phenomenon of emotion regulation in the context of psychosocial stress dynamics by investigating how emotion regulation mechanisms (including interoception, mindfulness, and cognitive reappraisal) are connected to EEG correlates of the psychosocial stress response. In order to achieve this goal, participants completed the FFMQ, MAIA, EROS, and IRI questionnaires and subsequently underwent the SST during which their EEG activity was continuously recorded.

Indeed, emotion regulation is typically studied using resting alpha frontal EEG asymmetry, but in accordance with Coen et al.’s [48] Capability model, state measures of neural correlates of emotion regulation might better explain behavioral variability during emotional challenges compared to trait measures. Therefore, we took into consideration every EEG band and interpreted the results on the basis of the existing literature.

Our findings mainly showed positive correlations between the psychometric measures of interoceptive awareness (MAIA) and mindfulness (FFMQ) and beta and gamma bands over frontal areas, as well as a positive correlation with the alpha band in temporo-central and occipital-parietal areas. On the other hand, positive correlations were also found between the psychometric measures of emotion regulation (EROS) and empathy (IRI) and delta, theta, and gamma in temporo-central and occipital-parietal areas.

Due to the substantial number of correlational findings, this discussion will focus primarily on the positive correlations deemed most relevant based on the existing literature. This selective approach enables a more concentrated exploration of the relationships that are likely to have significant implications for understanding the neural mechanisms underlying emotion regulation. By emphasizing these key correlations, the discussion will point out how specific aspects of interoception, mindfulness, emotion regulation, and empathy are reflected in distinct EEG patterns.

Firstly, concerning the results of MAIA and FFMQ, interoception and perception are central to understanding one’s own body stimuli and external stimuli, requiring a high level of awareness of bodily sensations and attention. Moreover, high levels of focused attention, attentional control, and regulation are necessary for both EA and IA. Interestingly, our results show positive correlations between FFMQ Observation and Acting with Awareness and MAIA Attention Regulation, Emotional Awareness, and Self-Regulation and both beta and gamma bands over frontal areas.

From a neuroscientific perspective, beta activity, particularly in the frontal region, has been associated with cognitive processes such as attention, self-regulation, and enhanced ability to focus and attend to bodily sensations during stressful situations [43]. Indeed, its increase during stress responses may signify ongoing cognitive processing, suggesting the activation of neural circuits to manage psychosocial stressors [44], thereby facilitating emotional awareness and self-regulation [51]. For this reason, the more beta activity increases in the frontal area, the higher the individual’s ability to concentrate and focus on their bodily state and sensations; this mechanism allows them to self-regulate thanks to the awareness of their emotional states, especially in stressful situations such as preparing and delivering a speech, during which negative emotional feedback is also provided. Thus, the increased beta power reflects ongoing cognitive processing and the activation of neural circuits to cope with stressors, underscoring its significance in facilitating adaptive responses to challenging environments.

On the other hand, gamma oscillations have been associated with various cognitive processes such as top-down attentional mechanisms, increased arousal, focused attention, and conscious awareness [46,54], and its increased activity in the frontal region has been correlated with enhanced focus on tasks and awareness of present sensations, which could be attributed to cognitive reappraisal and emotional regulation [57,63]. The role of the frontal cortex in emotion regulation and cognitive reappraisal [64,65] has been highlighted by functional magnetic resonance imaging (fMRI) studies that have shown how frontal cortex activity covaries with amygdala activity during reappraisal tasks, indicating the importance of frontal-amygdala connectivity in emotion regulation [66,67], whereas EEG studies have demonstrated that gamma band activity is closely related to the emotional processing and cognitive control of emotions [68,69]. 

Additionally, mindfulness training has been found to increase gamma band activity, reflecting improved emotional regulation [53] and, conversely, individuals with a greater capacity for cognitive restructuring after emotionally charged feedback and emotion regulation exhibit higher gamma activity [70]. For this reason, the more gamma activity increases in the frontal area, the higher the individual’s ability not only to focus on the task but also to be aware of the sensations experienced at that moment. Specifically, during the speech phase, this increase could be due to cognitive reappraisal following the feedback received (whether positive, negative, or neutral) and emotional regulation. Therefore, the more individuals can cognitively restructure their thoughts after feedback with a given emotional valence and regulate their emotions, the higher the gamma activity will be (as demonstrated by mindfulness studies).

Thirdly, our results also showed a positive correlation between MAIA Body Listening and the alpha band in TC and OP areas both during Pp and Sp. Alpha power has been consistently observed to decrease in response to psychosocial stress across various stress phases, which has been linked to increased cortical activity, reflecting an inhibitory control system in the brain that helps individuals cope with stressors. Indeed, psychosocial stress induces a state of heightened arousal and vigilance, activating the HPA axis and leading to greater cortical activity, which in turn reduces frontal alpha power [42]. Moreover, research suggests that individuals practicing mindfulness exhibit enhanced top-down control over sensory alpha, allowing for better regulation of cognitive performance, particularly in tasks requiring working memory [41,52]. By focusing on somatic sensations, mindfulness practitioners can modulate sensory alpha to suppress irrelevant stimuli and enhance internal attention, leading to improved cognitive processes [71].

For this reason, the higher the individual’s ability to actively listen to their body, the higher the alpha activity in the TC and OC areas will be. This likely indicates that individuals who can listen to and focus on their body not only exercise top-down control over sensory alpha, which enhances the ability to regulate cognitive performance, but also reduce rumination by using sensory alpha to suppress irrelevant and distracting internal stimuli (for example, ongoing negative ruminative memories or associations, in this case, due to feedback received during the speech) by paying attention to a sensory stimulus such as breathing.

Additional findings also showed a correlation between theta in PO areas and Act with Awareness and Delta in frontal and TC areas with MAIA Trusting. Both delta and theta oscillations are linked to emotional and motivational processes [38] critical for the body to respond to stressful situations. Particularly, during stressful events, delta waves are active in the frontal and temporo-central areas [39], supporting emotion management under pressure and coping mechanisms. Furthermore, delta waves have been correlated with autonomic regulation and deep rest, suggesting their role in promoting a sense of calmness and bodily trust [72]. Therefore, this physiological trust in bodily sensations, as indicated by the positive correlation with the “Trusting” subscale of MAIA, underscores their contribution to interoceptive perception and stress management.

On the other hand, theta waves, particularly in the parieto-occipital area, are critical for top-down regulation processes such as awareness, focused and sustained attention, inhibition and cognitive control, and problem-solving skills [38,40]. Therefore, theta activity’s positive correlation with the “Act with Awareness” subscale of FFMQ emphasizes the link between theta waves and mindfulness practices that enhance attentional capacities and meditative states. Moreover, theta waves have been associated with meditative states and reflective thinking, particularly useful during speech preparation and when reflecting on personal experiences [40]. This indicates that theta waves facilitate a cognitive state conducive to introspection and thoughtful response preparation.

Regarding the second set of results, IRI and EROS are related to the understanding of not only one’s own emotions but also those of others. This understanding requires a certain level of empathy, which has both an affective and a cognitive component, and it refers to both the emotional reaction of the individual and the ability to adopt another person’s perspective. Our findings showed positive correlations between (i) IRI Fantasy and delta, theta, and gamma in TC and OP areas; (ii) EROS Extrinsic and Intrinsic affective worsening and gamma in TC and PO areas; and (iii) EROS Intrinsic affective improving and alpha in TC areas.

Firstly, increased gamma activity in the posterior temporal area has been associated with a shift from narrative focus and self-referential processing towards heightened external attention on experiential focus and momentary sensory awareness and is believed to play a role in emotional regulation and is sensitive to individuals’ emotions [38]. Studies have also shown that spectral power in the gamma band and phase synchronization in specific brain regions, such as the left frontal and parietal regions, are linked to cognitive reappraisal following exposure to various stimuli [73], and functional connectivity in the low gamma range increases when individuals view distressing images [74]. Therefore, the higher the gamma activity over TC and OP areas, the higher the levels of affective worsening towards oneself and others, especially during the speech phase. This could be due to negative feedback received in previous speeches, which provokes negative feelings towards oneself and the examiners that the person is unable to suppress and regulate at that moment, given the stress and time pressure they are undergoing.

On the other hand, delta oscillations are hypothesized to be involved in motivational and emotional processes, potentially related to inhibiting sensory differences that could interfere with internal concentration [75], whereas Yao et al. [76] found that delta increased in a social judgment paradigm when participants received feedback in a social setting (reactive phase) as opposed to a non-social setting (control condition). Furthermore, theta frequency has been associated with various behavioral and emotional variables, as well as higher cognitive processes such as cognitive control, inhibition, and sustained attention [57]. This frequency band is known to play a crucial role in voluntary top-down regulation processes, with greater theta activity linked to increased awareness, clarity, acceptance, inhibitory control, and flexibility in emotion regulation strategies [57].

Therefore, the higher the tendency to empathize with fictional characters and feel pro-social emotions towards others, the higher the delta and theta activity. This could be due to motivational and emotional processes that, especially during the preparation phase, drive individuals to recall and empathize with situations they have already experienced, including the recall of the feelings that the situation evoked in others. This is particularly evident in D4, where the individual is asked to remember and describe a situation in which they had to make a decision alone, without being able to consult others, which involves empathizing with and imagining others’ reactions to that decision.

Finally, as previously stated, an alpha power reduction is observed in response to psychosocial stress, leading to heightened arousal and vigilance, along with the activation of the HPA axis [77]. This reduction in alpha power is indicative of increased cortical activity to effectively confront and adapt to stressors [77]. For this reason, since alpha decreases in response to stressful situations, the more an individual is able to deliberately improve their feelings, the more alpha activity increases, indicating the individual’s ability to manage and cope with the stressful situation effectively and consciously, also indicating greater stress tolerance.

In summary, this study highlighted the deep connection between EEG frequency bands and emotion-regulation strategies, which require high levels of awareness such as IA and EA. Overall, the results confirmed our hypothesis of positive correlations between the psychometric measures of interoceptive awareness (MAIA) and mindfulness (FFMQ) and the high-frequency bands (beta, alpha, and gamma), as well as the positive correlations between the psychometric measures of emotion regulation (EROS) and empathy (IRI) and the low-frequency bands (delta and theta). 

Overall, it is crucial to recognize a few limitations while evaluating the findings of this study. Firstly, the small sample size might affect the generalizability of findings; therefore, a larger sample size may be included in future research to achieve greater statistical robustness of the conclusions. Additionally, to further improve the validity and generalizability of the results, future studies should also strive for a more balanced representation of male and female participants, which is essential for identifying potential gender differences. Secondly, even though the SST mimics an interview situation from real life, the controlled laboratory environment might not fully capture the variety of stressors encountered in everyday life. Thirdly, even though EEG can provide a more in-depth understanding of the neural mechanisms underlying stress and emotional regulation, this study would benefit from incorporating additional physiological and psychological measures, such as hormonal analysis (e.g., cortisol levels) and recording of autonomic indices (e.g., heart rate and electrodermal activity) or self-report questionnaires assessing personality traits, as well as neuroimaging techniques like functional Near-Infrared Spectroscopy (fNIRS) to better understand the neural and metabolic brain activation to fully grasp the complexity of stress response. 

To conclude, this study represents a further step in understanding how stress and the way we manage and regulate it affects our brain activity and how the awareness of one’s body states is crucial in regulating stress and emotions in general to achieve an overall condition of well-being and better quality of life.

## Data Availability

The data presented in this study are available upon request from the corresponding author due to ethical reasons for sensitive personal data protection (requests will be evaluated according to the GDPR–Reg. UE 2016/679 and its ethical guidelines).

## References

[1] Mason J.W. (1975). A Historical View of the Stress Field. J. Hum. Stress..

[2] Chrousos G.P. (2009). Stress and Disorders of the Stress System. Nat. Rev. Endocrinol..

[3] Lazarus R.S. (1991). Emotion and Adaptation.

[4] Wang M., Saudino K.J. (2011). Emotion Regulation and Stress. J. Adult Dev..

[5] Gross J.J. (1998). Antecedent- and Response-Focused Emotion Regulation: Divergent Consequences for Experience, Expression, and Physiology. J. Pers. Soc. Psychol..

[6] Gross J.J., Thompson R.A., Gross J.J. (2007). Emotion Regulation: Conceptual Foundations. Handbook of Emotion Regulation.

[7] Brockman R., Ciarrochi J., Parker P., Kashdan T. (2017). Emotion Regulation Strategies in Daily Life: Mindfulness, Cognitive Reappraisal and Emotion Suppression. Cogn. Behav. Ther..

[8] Gross J.J., John O.P. (2003). Individual Differences in Two Emotion Regulation Processes: Implications for Affect, Relationships, and Well-Being. J. Pers. Soc. Psychol..

[9] Mauss I.B., Cook C.L., Cheng J.Y.J., Gross J.J. (2007). Individual Differences in Cognitive Reappraisal: Experiential and Physiological Responses to an Anger Provocation. Int. J. Psychophysiol..

[10] Gross J.J., Levenson R.W. (1993). Emotional Suppression: Physiology, Self-Report, and Expressive Behavior. J. Pers. Soc. Psychol..

[11] Balconi M., Molteni E. (2016). Past and Future of Near-Infrared Spectroscopy in Studies of Emotion and Social Neuroscience. J. Cogn. Psychol..

[12] Balconi M., Fronda G., Crivelli D. (2019). Effects of Technology-Mediated Mindfulness Practice on Stress: Psychophysiological and Self-Report Measures. Stress.

[13] Balconi M., Pozzoli U. (2005). Morphed facial expressions elicited a N400 ERP effect: A domain-specific semantic module?. Scand. J. Psychol..

[14] Zinn J.K. (1994). Wherever You Go, There You Are.

[15] Bishop S.R. (2002). What Do We Really Know about Mindfulness-Based Stress Reduction?. Psychosom. Med..

[16] Chambers R., Gullone E., Allen N.B. (2009). Mindful Emotion Regulation: An Integrative Review. Clin. Psychol. Rev..

[17] Angioletti L., Balconi M. (2020). Interoceptive Empathy and Emotion Regulation: The Contribution of Neuroscience. Neuropsychol. Trends.

[18] Zamariola G., Frost N., Van Oost A., Corneille O., Luminet O. (2019). Relationship between Interoception and Emotion Regulation: New Evidence from Mixed Methods. J. Affect. Disord..

[19] Hölzel B.K., Carmody J., Vangel M., Congleton C., Yerramsetti S.M., Gard T., Lazar S.W. (2011). Mindfulness Practice Leads to Increases in Regional Brain Gray Matter Density. Psychiatry Res. Neuroimaging.

[20] Pollatos O., Herbert B.M., Mai S., Kammer T. (2016). Changes in Interoceptive Processes Following Brain Stimulation. Philos. Trans. R. Soc. B Biol. Sci..

[21] Friedel S., Whittle S.L., Vijayakumar N., Simmons J.G., Byrne M.L., Schwartz O.S., Allen N.B. (2015). Dispositional Mindfulness Is Predicted by Structural Development of the Insula during Late Adolescence. Dev. Cogn. Neurosci..

[22] Farb N.A.S., Segal Z.V., Anderson A.K. (2013). Mindfulness Meditation Training Alters Cortical Representations of Interoceptive Attention. Soc. Cogn. Affect. Neurosci..

[23] Gibson J. (2019). Mindfulness, Interoception, and the Body: A Contemporary Perspective. Front. Psychol..

[24] Werner K., Gross J.J., Kring A.M., Sloan D.M. (2010). Emotion Regulation and Psychopathology: A Conceptual Framework. Emotion Regulation and Psychopathology: A Transdiagnostic Approach to Etiology and Treatment.

[25] Kever A., Pollatos O., Vermeulen N., Grynberg D. (2015). Interoceptive Sensitivity Facilitates Both Antecedent- and Response-Focused Emotion Regulation Strategies. Pers. Individ. Dif..

[26] Lane R.D., Schwartz G.E. (1987). Levels of Emotional Awareness: A Cognitive-Developmental Theory and Its Application to Psychopathology. Am. J. Psychiatry.

[27] Szczygieł D., Buczny J., Bazińska R. (2012). Emotion Regulation and Emotional Information Processing: The Moderating Effect of Emotional Awareness. Pers. Individ. Dif..

[28] Cameron O.G. (2001). Interoception: The inside Story—A Model for Psychosomatic Processes. Psychosom. Med..

[29] Barrett L.F., Bliss-Moreau E., Quigley K.S., Aronson K.R. (2004). Interoceptive Sensitivity and Self-Reports of Emotional Experience. J. Pers. Soc. Psychol..

[30] Mehling W.E., Price C., Daubenmier J.J., Acree M., Bartmess E., Stewart A. (2012). The Multidimensional Assessment of Interoceptive Awareness (MAIA). PLoS ONE.

[31] Baer R.A., Smith G.T., Lykins E., Button D., Krietemeyer J., Sauer S., Walsh E., Duggan D., Williams J.M.G. (2008). Construct Validity of the Five Facet Mindfulness Questionnaire in Meditating and Nonmeditating Samples. Assessment.

[32] Niven K., Totterdell P., Stride C.B., Holman D. (2011). Emotion Regulation of Others and Self (EROS): The Development and Validation of a New Individual Difference Measure. Curr. Psychol..

[33] Mayer R.C., Davis J.H., Schoorman F.D. (1995). An Integrative Model Of Organizational Trust. Acad. Manag. Rev..

[34] Vanhollebeke G., De Smet S., De Raedt R., Baeken C., van Mierlo P., Vanderhasselt M.A. (2022). The Neural Correlates of Psychosocial Stress: A Systematic Review and Meta-Analysis of Spectral Analysis EEG Studies. Neurobiol. Stress..

[35] Engert V., Efanov S.I., Duchesne A., Vogel S., Corbo V., Pruessner J.C. (2013). Differentiating Anticipatory from Reactive Cortisol Responses to Psychosocial Stress. Psychoneuroendocrinology.

[36] McEwen B.S. (2007). Physiology and Neurobiology of Stress and Adaptation: Central Role of the Brain. Physiol. Rev..

[37] McEwen B.S., Gianaros P.J. (2011). Stress- and Allostasis-Induced Brain Plasticity. Annu. Rev. Med..

[38] Knyazev G.G. (2007). Motivation, Emotion, and Their Inhibitory Control Mirrored in Brain Oscillations. Neurosci. Biobehav. Rev..

[39] Tóth B., Farkas D., Urbán G., Szalárdy O., Orosz G., Hunyadi L., Hajdu B., Kovács A., Szabó B.T., Shestopalova L.B. (2019). Attention and Speech-Processing Related Functional Brain Networks Activated in a Multi-Speaker Environment. PLoS ONE.

[40] Craig A.D., Rd T. (2011). Significance of the Insula for the Evolution of Human Awareness of Feelings from the Body. Ann. N. Y. Acad. Sci..

[41] Haegens S., Händel B.F., Jensen O. (2011). Top-Down Controlled Alpha Band Activity in Somatosensory Areas Determines Behavioral Performance in a Discrimination Task. J. Neurosci..

[42] Campbell J., Ehlert U. (2012). Acute Psychosocial Stress: Does the Emotional Stress Response Correspond with Physiological Responses?. Psychoneuroendocrinology.

[43] Sauseng P., Hoppe J., Klimesch W., Gerloff C., Hummel F.C. (2007). Dissociation of Sustained Attention from Central Executive Functions: Local Activity and Interregional Connectivity in the Theta Range. Eur. J. Neurosci..

[44] Liao Y.C., Guo N.W., Su B.Y., Chen S.J., Tsai H.F., Lee K.Y. (2020). Frontal Beta Activity in the Meta-Intention of Children With Attention Deficit Hyperactivity Disorder. Clin. EEG Neurosci..

[45] Kang J.H., Ahn H.M., Jeong J.W., Hwang I., Kim H.T., Kim S.H., Kim S.P. (2012). The Modulation of Parietal Gamma Oscillations in the Human Electroencephalogram with Cognitive Reappraisal. Neuroreport.

[46] Ochsner K.N., Bunge S.A., Gross J.J., Gabrieli J.D.E. (2002). Rethinking Feelings: An FMRI Study of the Cognitive Regulation of Emotion. J. Cogn. Neurosci..

[47] Yang K., Tong L., Shu J., Zhuang N., Yan B., Zeng Y. (2020). High Gamma Band EEG Closely Related to Emotion: Evidence From Functional Network. Front. Hum. Neurosci..

[48] Coan J.A., Allen J.J.B., McKnight P.E. (2006). A Capability Model of Individual Differences in Frontal EEG Asymmetry. Biol. Psychol..

[49] Allen A.P., Kennedy P.J., Dockray S., Cryan J.F., Dinan T.G., Clarke G. (2017). The Trier Social Stress Test: Principles and Practice. Neurobiol. Stress..

[50] Kirschbaum C., Pirke K.M., Hellhammer D.H. (1993). The “Trier Social Stress Test”-A Tool for Investigating Psychobiological Stress Responses in a Laboratory Setting. Neuropsychobiology.

[51] Laufs H., Krakow K., Sterzer P., Eger E., Beyerle A., Salek-Haddadi A., Kleinschmidt A. (2003). Electroencephalographic Signatures of Attentional and Cognitive Default Modes in Spontaneous Brain Activity Fluctuations at Rest. Proc. Natl. Acad. Sci. USA.

[52] Waldhauser G.T., Lindgren M., Johansson M. (2012). Intentional Suppression Can Lead to a Reduction of Memory Strength: Behavioral and Electrophysiological Findings. Front. Psychol..

[53] Banks S.J., Eddy K.T., Angstadt M., Nathan P.J., Luan Phan K. (2007). Amygdala–Frontal Connectivity during Emotion Regulation. Soc. Cogn. Affect. Neurosci..

[54] Kang J.H., Jeong J.W., Kim H.T., Kim S.H., Kim S.P. (2014). Representation of Cognitive Reappraisal Goals in Frontal Gamma Oscillations. PLoS ONE.

[55] Hogeveen J., Salvi C., Grafman J. (2016). ‘Emotional Intelligence’: Lessons from Lesions. Trends Neurosci..

[56] Busselle R., Bilandzic H. (2009). Measuring Narrative Engagement. Media Psychol..

[57] Haaf M., Polomac N., Starcevic A., Lack M., Kellner S., Dohrmann A.-L., Fuger U., Steinmann S., Rauh J., Nolte G. (2024). Frontal Theta Oscillations during Emotion Regulation in People with Borderline Personality Disorder. BJPsych Open.

[58] Cohen S., Kamarck T., Mermelstein R. (1983). A Global Measure of Perceived Stress. J. Health Soc. Behav..

[59] Giovannini C., Giromini L., Bonalume L., Tagini A., Lang M., Amadei G. (2014). The Italian Five Facet Mindfulness Questionnaire: A Contribution to Its Validity and Reliability. J. Psychopathol. Behav. Assess.

[60] Jones A., Silas J., Todd J., Stewart A., Acree M., Coulson M., Mehling W.E. (2021). Exploring the Multidimensional Assessment of Interoceptive Awareness in Youth Aged 7–17 Years. J. Clin. Psychol..

[61] Mehling W.E., Acree M., Stewart A., Silas J., Jones A. (2018). The Multidimensional Assessment of Interoceptive Awareness, Version 2 (MAIA-2). PLoS ONE.

[62] Albiero P., Ingoglia S., Lo Coco A. (2006). Contribute All’adattamento Italiano Dell’Interpersonal Reactivity Index. Test. Psicometria Metodol..

[63] Oathes D.J., Ray W.J., Yamasaki A.S., Borkovec T.D., Castonguay L.G., Newman M.G., Nitschke J. (2008). Worry, Generalized Anxiety Disorder, and Emotion: Evidence from the EEG Gamma Band. Biol. Psychol..

[64] Cartocci G., Giorgi A., Inguscio B.M.S., Scorpecci A., Giannantonio S., De Lucia A., Garofalo S., Grassia R., Leone C.A., Longo P. (2021). Higher Right Hemisphere Gamma Band Lateralization and Suggestion of a Sensitive Period for Vocal Auditory Emotional Stimuli Recognition in Unilateral Cochlear Implant Children: An EEG Study. Front. Neurosci..

[65] Li W., Zhang W., Jiang Z., Zhou T., Xu S., Zou L. (2022). Source Localization and Functional Network Analysis in Emotion Cognitive Reappraisal with EEG-FMRI Integration. Front. Hum. Neurosci..

[66] Pierce J.E., Blair R.J.R., Clark K.R., Neta M. (2022). Reappraisal-Related Downregulation of Amygdala BOLD Activation Occurs Only during the Late Trial Window. Cogn. Affect. Behav. Neurosci..

[67] De la Peña-Arteaga V., Morgado P., Couto B., Ferreira S., Castro I., Sousa N., Soriano-Mas C., Picó-Pérez M. (2022). A Functional Magnetic Resonance Imaging Study of Frontal Networks in Obsessive-Compulsive Disorder during Cognitive Reappraisal. Eur. Psychiatry.

[68] Dougherty L.R., Blankenship S.L., Spechler P.A., Padmala S., Pessoa L. (2015). An FMRI Pilot Study of Cognitive Reappraisal in Children: Divergent Effects on Brain and Behavior. J. Psychopathol. Behav. Assess.

[69] Hiebler-Ragger M., Perchtold-Stefan C.M., Unterrainer H.F., Fuchshuber J., Koschutnig K., Nausner L., Kapfhammer H.P., Papousek I., Weiss E.M., Fink A. (2021). Lower Cognitive Reappraisal Capacity Is Related to Impairments in Attachment and Personality Structure in Poly-Drug Use: An FMRI Study. Brain Imaging Behav..

[70] Razavi M.S., Tehranidoost M., Ghassemi F., Purabassi P., Taymourtash A. (2017). Emotional Face Recognition in Children With Attention Deficit/Hyperactivity Disorder: Evidence From Event Related Gamma Oscillation. Basic. Clin. Neurosci..

[71] Kerr N.L., Tindale R.S. (2011). Group-Based Forecasting?: A Social Psychological Analysis. Int. J. Forecast..

[72] Haslacher D., Reber P., Cavallo A., Rosenthal A., Pangratz E., Beck A., Romanczuk-Seiferth N., Nikulin V., Villringer A., Soekadar S.R. (2024). Heartbeat Perception Is Causally Linked to Frontal Delta Oscillations. bioRxiv.

[73] Dixon M.L., Thiruchselvam R., Todd R., Christoff K. (2017). Emotion and the Prefrontal Cortex: An Integrative Review. Psychol. Bull..

[74] Koole S.L. (2009). The Psychology of Emotion Regulation: An Integrative Review. Cogn. Emot..

[75] Herrmann C.S., Strüber D., Helfrich R.F., Engel A.K. (2016). EEG Oscillations: From Correlation to Causality. Int. J. Psychophysiol..

[76] Yao Y., Wu M., Wang L., Lin L., Xu J. (2020). Phase Coupled Firing of Prefrontal Parvalbumin Interneuron With High Frequency Oscillations. Front. Cell Neurosci..

[77] Johnson J.M., Durrant S.J. (2018). The Effect of Cathodal Transcranial Direct Current Stimulation during Rapid Eye-Movement Sleep on Neutral and Emotional Memory. R. Soc. Open Sci..

